# Polymorphism of *NCAPG* gene and its association with growth traits in Nanjiang Yellow goat

**DOI:** 10.1080/10495398.2025.2565161

**Published:** 2025-10-07

**Authors:** Guolin Chen, Tao Ma, Jing Luo, Mancheng Zhang, Shun Wu, Yu Chen, Yong Chen, Deji Zhuoga, Yuan Chen, He Cai, Hongping Zhang, Jiazhong Guo, Jiaxue Cao, Dinghui Dai, Li Li

**Affiliations:** ^a^ College of Animal Science and Technology, Sichuan Agricultural University, Chengdu, China; ^b^ Mianyang Academy of Agricultural Sciences, Mianyang, China; ^c^ Institute of Nanjiang Yellow Goat Breeding Science, Nanjiang, China; ^d^ Kamba Town Agricultural and Pastoral Comprehensive Service Center of Gamba County, Shigatse, China

**Keywords:** Nanjiang yellow goat, NCAPG, SNP, growth traits, association analysis

## Abstract

The growth of livestock is influenced by both genetic and environmental factors, with genes like Non-SMC Condensin I complex subunit G (*NCAPG*) playing key roles in regulating growth. Previous studies have suggested that *NCAPG* is associated with growth traits in species such as cattle, sheep, and chickens. In this study, we comprehensively assessed *NCAPG* polymorphisms and their association with body weight (BW), chest circumference (CC), body height (BH), and body length (BL) at six stages (from newborn to 30 months) in 469 Nanjiang Yellow goats, utilizing the Generalized Linear Mixed Models (GLMM). Pearson correlation analysis revealed the strongest correlation between BW and CC (R = 0.91), followed by BW and BL (R = 0.87), and BW and BH (R = 0.89). Additionally, 24 single nucleotide polymorphisms (SNPs) within the NCAPG gene were identified, which were relatively conserved across closely related species. A notable missense mutation (g.5705 A > G) in exon 6 resulted in an amino acid substitution (281 Ile > Met), which may alter the physicochemical properties and conformation of the NCAPG protein. The resulting impairment in condensin function delays chromosome segregation, prolongs metaphase in growth plate stem cells, and enhances proliferative signaling, ultimately leading to variations in body size. Correlation analysis revealed that 13 of these SNPs were concurrently associated with multiple growth traits, including BH, BL, and CC. These polymorphisms in the *NCAPG* gene represent potential candidates for marker-assisted selection (MAS) to improve body size in Nanjiang Yellow goats.

## Introduction

The growth of livestock results from the combined influence of genetic and environmental factors.^1^ Superior growth traits typically lead to increased production efficiency and economic benefits, thereby boosting the incomes of farmers.^2^ The Nanjiang Yellow goat, a meat-type breed, is renowned for its excellent meat production performance. As an artificially selected breed, it possesses key characteristics such as high meat quality, strong adaptability, rapid growth and development, stable genetic traits, and high reproductive capability.^3^^,^^4^ However, significant variation in body size is observed among individuals. Among the primary economic traits of goats, growth traits are crucial for optimizing and expanding meat goat production on a large scale.^5^ Therefore, understanding the genetics underlying these traits is crucial for their genetic improvement, which relies significantly on the efficient molecular markers associated with superior growth characteristics.^6^

Non-SMC Condensin I complex subunit G (*NCAPG*), a mitosis-associated chromosomal condensation protein, is crucial for maintaining the structure of chromosomes.^7^^,^^8^ The structural stability of chromosomes is vital for normal organism development and life activities,^9–12^ and numerous researchers have delved into the intricate association between *NCAPG* and various forms of cancer.^11^^,^^13–15^ These studies underscore the ubiquitous upregulation of *NCAPG* across diverse cancer types, highlighting its pivotal role in tumor cell proliferation, invasion, metastasis, and apoptosis.

Beyond its role in cancer biology, *NCAPG* has attracted attention in livestock genetics. Genome-wide association studies (GWAS) and selection signature analyses have identified *NCAPG* as a potential candidate gene for growth traits in cattle, horses, donkeys, sheep, and goats,^16–23^ with significant associations reported for muscle growth, body size, average daily gain, and carcass weight in cattle.^22–26^ However, compared with these species, studies on the genetic variations of *NCAPG* in goats remain limited, and the potential mechanisms linking *NCAPG* polymorphisms to growth traits in goats are still poorly understood.

To address this gap, the present study focuses on SNPs within the NCAPG gene of Nanjiang Yellow goats, aiming to predict their effects on protein function and to investigate their association with growth traits. This work provides novel insights into the role of NCAPG in caprine growth and offers a potential basis for developing molecular markers to facilitate genetic improvement in goats.

## Materials and methods

### Collection of blood samples and phenotype data

A total of 469 healthy individuals were sampled, consisting of 205 males and 264 females from the Nanjiang Yellow Goat Breeding Farm in Nanjiang County, Sichuan Province, China. These individuals were subjected to consistent husbandry and management practices. All goats had recorded birth weights (BW0) and were maintained under standardized feeding and environmental conditions. Subsequently, at predetermined 2 months (M2), 4 months (M4), 6 months (M6), 12 months (M12), and 30 months (M30), comprehensive phenotypic assessments were conducted, including body weight (BW), body height (BH), body length (BL), and chest circumference (CC). To ensure data reliability, all measurements were performed by the same trained personnel using calibrated instruments, and each measurement was taken in duplicate. Average values were recorded for analysis to minimize operator bias and technical variability.

Blood samples were aseptically collected via jugular venipuncture, utilizing EDTA-2K as an anticoagulant, and subsequently stored at −20 °C for future analyses. Standard operating procedures were followed throughout sampling and handling to maintain consistency and sample integrity.

### DNA quality detection and construction of the mixed Pool

The genomic DNA (gDNA) was extracted from these blood samples using a commercial blood DNA extraction kit that was bought from Tiangen Biochemical Technology Co., Ltd. in Beijing, China. The concentration and purity of DNA extraction were assessed using the NanoDrop 2000 spectrophotometer. DNA samples were deemed eligible if their concentration was greater than 50 ng/μL and their OD260/OD280 ratio was between 1.8 and 2. The qualified gDNA was uniformly diluted to 20 ng/μL. A gDNA pool was conducted by randomly selecting 30 samples (15 males and 15 females) with 5 μL from each.

### PCR amplification, sequencing and genotyping

Based on our laboratory previous whole-genome resequencing data, we identified 24 candidate single nucleotide polymorphisms (SNPs) in *NCAPG* gene of the Nanjiang Yellow goat.^27^ Specific primers targeting these 24 SNPs were designed using Primer 5.0 software^28^ based on the *NCAPG* gene sequence (>NC_030813.1:37858058-37903119 Capra hircus breed San Clemente chromosome 6, ASM170441v1 Gene ID: 102176682; Figure S1) and synthesized by Beijing Qingke Biotechnology Co., Ltd. The following were included in the amplification system: 15 μL 2 × Taq PCR Master mix, 2 μL template, 1.5 μL of each upstream and downstream primer, and 10 μL ultrapure water. Then, PCR amplification was carried out using the mixed gDNA as a template, and the amplified products were sent to Beijing Qingke Biotechnology Co., Ltd. for Sanger sequencing. The PCR amplification primers are listed in Table S1. The amplification system included 15 μL 2 × Taq PCR Master mix, 2 μL template, upstream and downstream primer, 1.5 μL each, and 10 μL ultrapure water. The PCR protocol was set up as follows: 95 °C for 5 minutes, followed by 36 cycles of 95 °C for 30 seconds, annealing for 30 seconds (Table S1 provides the annealing temperatures), 72 °C for 2 minutes, extension at 72 °C for 7 minutes, and finally, 4 °C insulation. BioEdit v7.2.5.0 software^29^ was used to compare the sequencing findings in order to confirm the SNPs that were previously found using whole genome resequencing.

The gDNA from 469 individuals was provided to Bomiao Biotechnology Co., Ltd (Beijing), where high-throughput genotyping of the aforementioned 24 SNPs was conducted using the Agena Bioscience MassARRAY system with the corresponding 1st-PCRP, 2nd-PCRP, and UEP—SEQ for multiple SNPs designed by Assay Designer 4.0 (Agena Bioscience) (Table S2).

### Structure prediction and physicochemical properties analysis of NCAPG protein altered by SNP

We determined the amino acid composition of goat NCAPG protein (Accession: XM_005681456.3, containing 1,017 amino acid residues) using PredictProtein (https://predictprotein.org/). Additionally, the Expasy (https://web.expasy.org/) was used to assess the physicochemical characteristics of the NCAPG protein, and phyre2 (http://www.sbg.bio.ic.ac.uk/phyre2/) and I-TASSER (https://zhanggroup.org/I-TASSER/) were used to predict the protein structure. In the meantime, the glycosylation and phosphorylation sites of the NCAPG protein were predicted using NetPhos-3.1 (NetPhos 3.1 - DTU Health Tech - Bioinformatic Services) and DictyOGlyc-1.1 (DictyOGlyc 1.1 - DTU Health Tech - Bioinformatic Services) in DTU Health Tech Services (https://services.healthtech.dtu.dk/services/), and IMu-tant (https://folding.biofold.org/i-mutant/i-mutant2.0.html) was used to assess stability of the protein.

### Data statistical analysis

Using the algorithm in Microsoft Excel, we calculated genotype frequency, allele frequency, effective allele frequency, heterozygosity, homozygosity, Hardy-Weinberg equilibrium (HWE), and polymorphism information content (PIC) of the *NCAPG* gene. The chi-square test was used to perform initial population genetic assessments (e.g., Hardy–Weinberg equilibrium). Moreover, Haploview software^30^ was employed to analyse the linkage between SNP loci and construct a linkage map. We employed the Generalized Linear Mixed Models (GLMM) model in SAS v9.4 software^31^ with the following model formula to measure the relationship between the *NCAPG* gene and growth attributes in Nanjiang Yellow goats (supplementary section). The model aims to assess the impact of genotype, sex, season, and growth stage (time) on the dependent variable y, with both main effects and interactions considered. The random effects ($\gamma_{i}$) account for unobserved heterogeneity, and the residual error ($e_{i}$) captures unexplained variation. For multiple testing correction, we applied false discovery rate control (Benjamini-Hochberg procedure) at q < 0.05.

## Results

### Correlation of growth traits in Nanjiang Yellow goat

**Figure 1.**
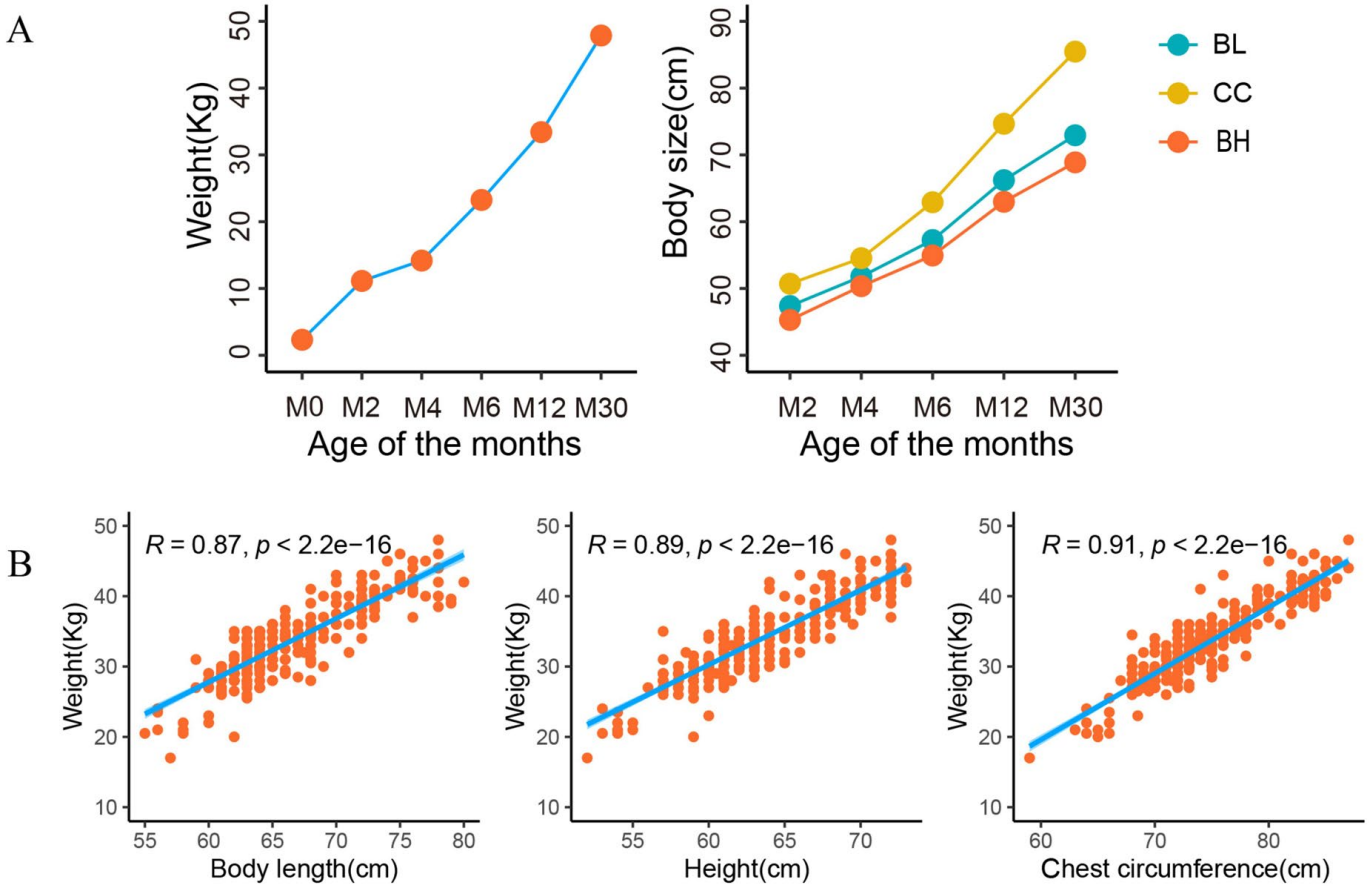
The growth and development of Nanjiang Yellow goat. A: Growth curve of body size and body weight. B: Correlation between body size and body weight. BL: Body length, CC: Chest circumference, BH: Body height.

The correlation analysis body weight (BW) and body size (containing BH, BL, and CC) of the Nanjiang Yellow goat was done using growth curves and Pearson correlation coefficients. The study revealed a consistent growth pattern in both body weight and morphometric traits among Nanjiang Yellow goats, demonstrating a significant positive correlation with advancing age from birth until 30 months (Figure 1(A)). The correlation coefficients (R) between BL, BH, and CC, with BW were 0.87, 0.89, and 0.91, respectively (Figure 1(B)). This suggests that CC was the most linked with BW and might be a useful indication for BW estimation.

### Polymorphism of NCAPG gene in Nanjiang Yellow goat

**Figure 2.**
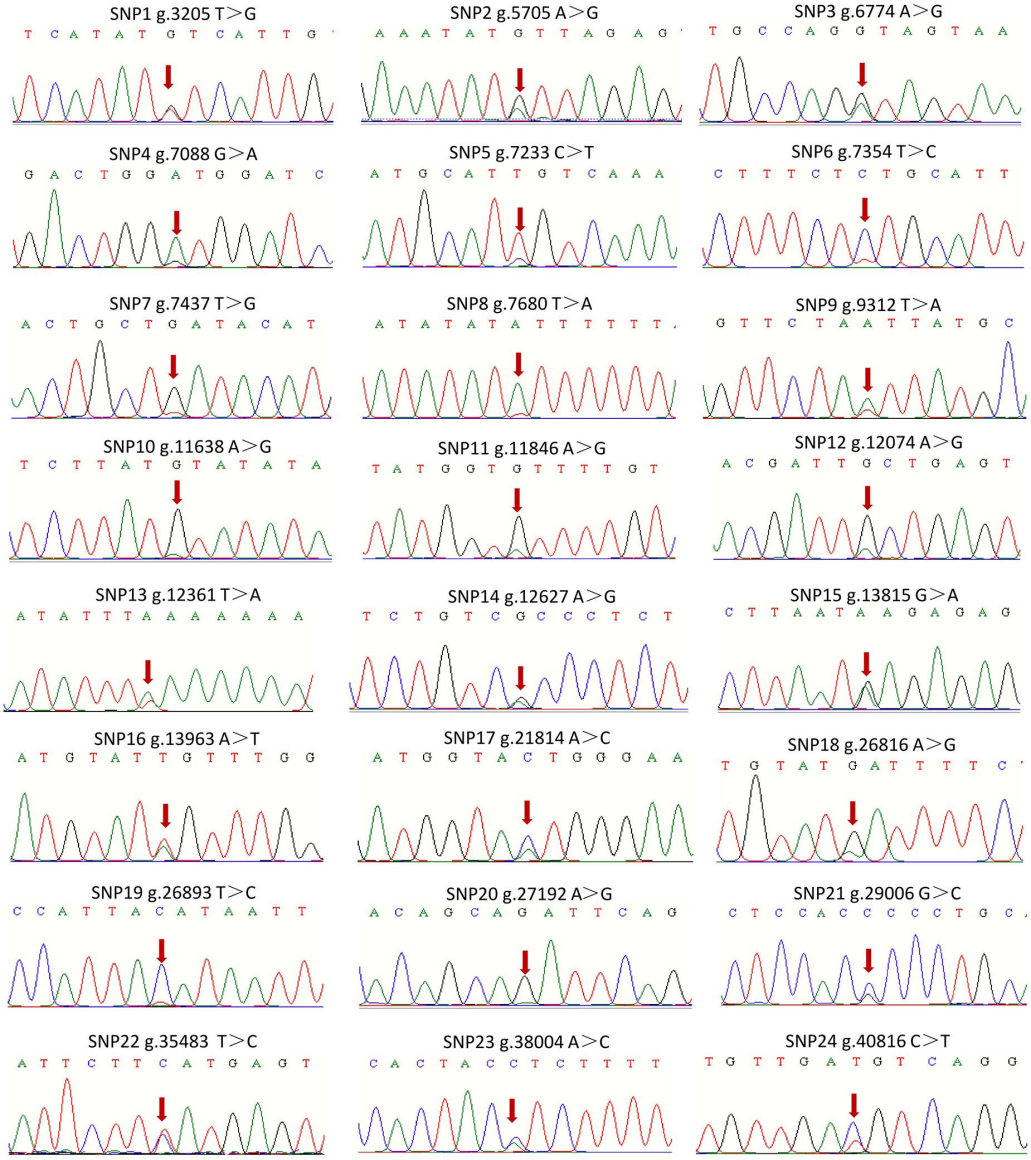
SNPs Of *NCAPG* gene identified in Nanjiang yellow goats. Red arrows pointed the mutated nucleotides.

**Figure 3.**
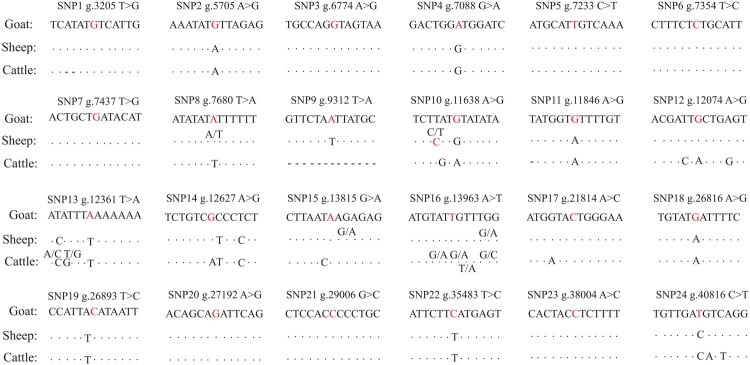
Comparison alignment of 24 *NCAPG* gene SNPs of Nanjiang Yellow goat with sheep and cattle genomic variations. Red indicates mutation sites, while dashes (-) represent positions where alignment with the reference sequence was not achieved.

We identified 24 SNPs within the *NCAPG* gene of Nanjiang Yellow goats (Figure 2) using validated gDNA templates (Figure S1) and PCR-Sanger sequencing. Among these, 22 SNPs were located in intronic regions, while 2 were found in exonic regions (g.5705 A > G and g.12074 A > G) (Table 1, Tables S1–S2, Figure 2). Comparative analysis with genomic variations in sheep and cattle revealed that these 24 SNPs in Nanjiang Yellow goats exist as either wild-type or mutant alleles in closely related species. Notably, sheep exhibited the same mutation at the g.7680 T > A locus (Figure 3). Across the goat population, observed and expected heterozygosity ranged from 0.037 to 0.736 and 0.036 to 0.500, respectively, while polymorphism information content (PIC) values ranged from 0.035 to 0.375. As only one genotype (AG) was identified at locus g.12627 A > G, no further analyses were performed for this site. Of the remaining 23 SNPs, 20 exhibited moderate polymorphism (0.25 < PIC ≤ 0.5), and 3 exhibited low polymorphism (PIC ≤ 0.25). Furthermore, 19 SNPs conformed to Hardy–Weinberg equilibrium (P > 0.05), while 4 SNPs (g.7088 G > A, g.9312 T > A, g.11846 A > G, and g.12361 T > A) deviated from equilibrium, suggesting they are under selection (P < 0.05) (Table 1).

**Table 1.** Location and amino acid changes of *NCAPG* SNPs in Nanjiang Yellow goat.

Locus	Position	Amino acid change	No. samples	Genotypic frequency			Allele frequency		Observed heterozygosity(Ho)	Expected heterozygosity(HE)	χ^2^(HWE)	P-value	Polymorphism information content(PIC)
g.12074 a > G	Exon9	No	465	AA(0.073)	AG(0.342)	GG(0.585)	A(0.244)	G(0.756)	0.342	0.369	2.309	P > 0.05	0.301
g.3205 T > G	Intron3	No	465	GG(0.142)	GT(0.419)	TT(0.439)	G(0.352)	T(0.648)	0.419	0.456	2.939	P > 0.05	0.352
g.5705 A > G	Exon6	Ile (I)>Met (M)	469	AA(0.486)	GA(0.416)	GG(0.098)	A(0.694)	G(0.306)	0.416	0.425	0.204	P > 0.05	0.335
g.6774 A > G	Intron7	No	466	AA(0.073)	AG(0.358)	GG(0.569)	A(0.252)	G(0.748)	0.358	0.377	1.093	P > 0.05	0.306
g.7088 G > A	Intron7	No	466	AA(0.524)	GA(0.354)	GG(0.122)	A(0.701)	G(0.299)	0.354	0.419	10.657	p < 0.05	0.332
g.7233 C > T	Intron7	No	466	TT(0.910)	TC(0.090)		T(0.955)	C(0.045)	0.090	0.086	0.087	P > 0.05	0.082
g.7354 T > C	Intron7	No	466	CC(0.573)	CT(0.354)	TT(0.073)	C(0.750)	T(0.250)	0.354	0.375	1.364	P > 0.05	0.305
g.7437 T > G	Intron7	No	466	GG(0.569)	GT(0.358)	TT(0.073)	G(0.748)	T(0.252)	0.358	0.377	1.093	P > 0.05	0.306
g.7680 T > A	Intron7	No	468	AA(0.613)	TA(0.338)	TT(0.049)	A(0.782)	T(0.218)	0.338	0.342	0.043	P > 0.05	0.283
g.9312 T > A	Intron7	No	466	AA(0.534)	TA(0.343)	TT(0.122)	A(0.706)	T(0.294)	0.343	0.415	13.021	p < 0.05	0.329
g.11638 A > G	Intron8	No	429	AA(0.077)	AG(0.35)	GG(0.573)	A(0.252)	G(0.748)	0.350	0.377	2.061	P > 0.05	0.306
g.11846 A > G	Intron8	No	466	AA(0.122)	GA(0.736)	GG(0.536)	A(0.293)	G(0.707)	0.736	0.414	13.523	p < 0.05	0.328
g.12361 T > A	Intron9	No	469	AA(0.527)	AT(0.351)	TT(0.126)	A(0.700)	T(0.300)	0.351	0.420	13.021	p < 0.05	0.332
g.12627 A > G	Intron9	No	469	AG(1.000)			A(0.500)	G(0.500)					
g.13815 G > A	Intron9	No	466	AA(0.571)	GA(0.357)	GG(0.073)	A(0.749)	G(0.251)	0.357	0.376	1.225	P > 0.05	0.305
g.13963 A > T	Intron9	No	467	AA(0.075)	AT(0.361)	TT(0.565)	A(0.255)	T(0.745)	0.361	0.380	1.228	P > 0.05	0.308
g.21814 A > C	Intron12	No	469	AA(0.047)	CA(0.325)	CC(0.631)	A(0.208)	C(0.792)	0.325	0.329	0.226	P > 0.05	0.275
g.26816 A > G	Intron13	No	466	AA(0.657)	AG(0.301)	GG(0.043)	A(0.807)	G(0.193)	0.301	0.312	0.561	P > 0.05	0.263
g.26893 T > C	Intron13	No	444	CC(0.903)	CT(0.097)		C(0.952)	T(0.048)	0.097	0.092	0.104	P > 0.05	0.088
g.27192 A > G	Intron13	No	469	GG(0.963)	GA(0.037)		G(0.982)	A(0.018)	0.037	0.036	0.006	P > 0.05	0.035
g.29006 G > C	Intron14	No	466	CC(0.258)	CG(0.484)	GG(0.26)	C(0.499)	G(0.501)	0.484	0.500	0.550	P > 0.05	0.375
g.35483 T > C	Intron14	No	466	CC(0.135)	TC(0.443)	TT(0.423)	C(0.356)	T(0.644)	0.443	0.459	0.604	P > 0.05	0.353
g.38004 A > C	Intron14	No	466	AA(0.009)	CA(0.161)	CC(0.83)	A(0.089)	C(0.911)	0.161	0.162	0.028	P > 0.05	0.149
g.40816 C > T	Intron16	No	466	CC(0.427)	TC(0.432)	TT(0.142)	C(0.643)	T(0.357)	0.432	0.459	1.699	P > 0.05	0.354

Note: χ^2^_0.05(2)_=5.991, χ^2^_0.01(2)_ =9.2.

Additionally, 23 SNPs were organized into three linkage disequilibrium (LD) blocks (blocks 1, 2, and 3), comprising 11, 4, and 4 SNPs, respectively. Four SNPs were not incorporated into any LD block (Table 2, Figure S2). The correlation coefficient between block 1 and block 2 was 1.0, indicating complete linkage, while the correlation coefficient between block 2 and block 3 was 0.93 (Figure S2). Moreover, 11, 7, and 11 combined genotypes were identified in blocks 1, 2, and 3, respectively (Table 4). The most frequent genotypes were GAGGAACCGGAAGGGGG (27.42%) in block 1, AATTCCAA (56.80%) in block 2, and CGTCCCTC (26.50%) in block 3, whereas the least frequent genotypes were AAAGGACTGTTAGGGG (0.94%), GGAAAAAG (0.43%), and CCTTAACC (0.80%) in blocks 1, 2, and 3, respectively (Table 4).

### The effect of NCAPG mutation g.5705 a > G on the protein

Of the two SNPs identified in exons, g.12074 A > G in exon 9 was a synonymous mutation, whereas g.5705 A > G in exon 6 altered the codon from ATA to ATG, resulting in an amino acid substitution from Isoleucine (Ile, NCAPG-281I) to Methionine (Met, NCAPG-281M) (Table 1). This substitution slightly decreased the proportion of Ile (from 6.50% to 6.40%) and increased the proportion of Met (from 2.10% to 2.20%) in the NCAPG protein (Table S3). Compared with the wild-type g.5705 A (NCAPG-281I), the mutant allele g.5705 G (NCAPG-281M) led to a modest reduction in the total number of atoms, instability index, aliphatic index, and GRAVY score of the NCAPG protein (Table S4, Figure S3). The NCAPG-281I > M substitution was located within the YCG1 (Interval 5–826) and PDS5 (Interval 99–359) domains (Figure S1), suggesting potential structural effects. Protein stability was reduced, as indicated by a negative change in free energy (ΔΔG = −0.826). The g.5705 A > G mutation did not substantially affect the glycosylation or phosphorylation of the protein (Figure S4).

**Figure 4.**
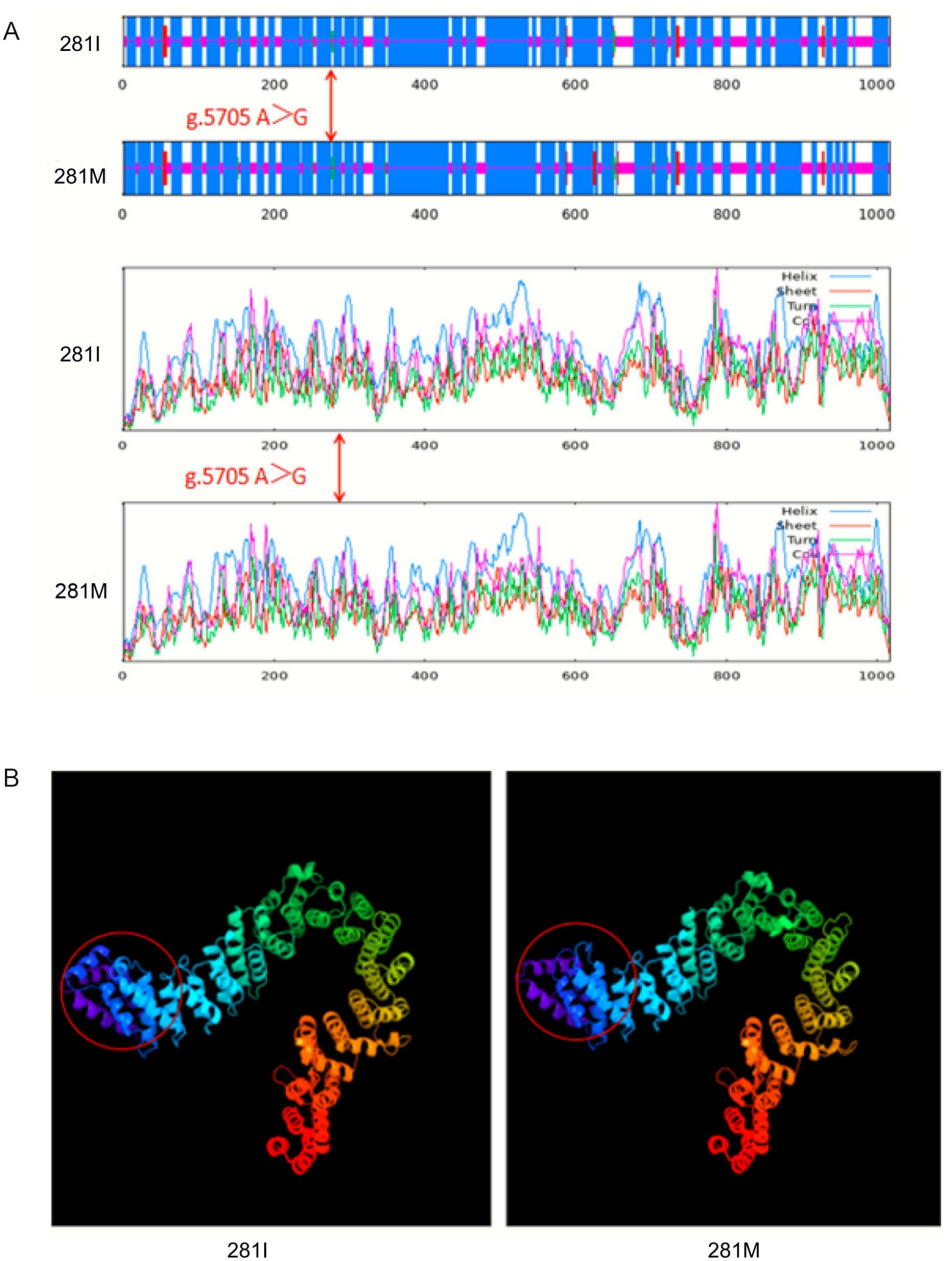
The protein structure prediction of NCAPG-281I and NCAPG-281M. A: Secondary structure prediction of NCAPG-281I and NCAPG-281M. B: Tertiary structure prediction of NCAPG-281I and NCAPG-281M. Note: the red circle represents the area of structural change. Note: At the same block, different superscript lowercase letters represent P < 0.05, and the same letter or no letters represent P > 0.05.

Moreover, the secondary structure of the NCAPG protein was altered by g.5705 A > G mutation (Figure 4(A)). The composition characteristics of NCAPG-281I showed that Alpha helix (Hh), Extended strand (Ee), β-turn (Tt), and Random coil (Cc) account for 69.71%, 1.18%, 0.88%, and 28.22%, respectively; These were altered in NCAPG-281M (Hh 69.52%, Ee 1.47%, Tt 0.98%, and Cc 28.02%) (Table S5). Along with this, the regions with amino acid alterations in the tertiary structure of the NCAPG-281I protein differ from those in the NCAPG-281M protein (Figure 4(B)).

### Association of NCAPG SNPs with growth traits in Nanjiang Yellow goat

Based on the GLMM analysis, SNPs in the *NCAPG* gene of Nanjiang Yellow goats showed no significant overall association with BW0 and average daily gain (Table S6 and Table 2). However, 19 of the 23 SNPs exhibited significant associations with body size traits at specific developmental stages. In particular, 18 SNPs were associated with BH (*p* < 0.05), 17 with BL (*p* < 0.05), and 13 with CC (*p* < 0.05) (Table S6), with 10 SNPs concurrently influencing all three traits. Age-dependent effects were also observed, as no significant associations were detected for BW0, BW or BL at 4 months, or for BH, BL, and CC at 6 months (Table S6). Moreover, several SNPs exhibited stage-specific effects, such as g.7233 C > T, which was linked to BL only at 30 months, and g.7680 T > A, which was associated with BH at 4 months (Table S6). These results highlight the age- and trait-specific contributions of *NCAPG* polymorphisms to growth regulation in goats.

**Table 2.** Association analysis of *NCAPG* SNPs genotype with body size in Nanjiang Yellow goat.

Locus	Genotype(N)	Average daily gain/g	Body height/cm	Body length/cm	Chest circumference/cm	Block
g.3205 T > G	GG(66)	51.81 ± 0.961	56.07 ± 1.25^b^	59.14 ± 1.61	65.37 ± 2.08	–
	GT(195)	53.21 ± 0.710	56.68 ± 1.24^a^	59.41 ± 1.60	65.89 ± 2.07	–
	TT(204)	51.53 ± 0.670	56.51 ± 1.24^ab^	59.11 ± 1.60	65.59 ± 2.07	–
g.5705 A > G	AA(228)	51.19 ± 0.624	56.40 ± 1.20	59.06 ± 1.60^b^	65.51 ± 2.05	Block 1
	GA(195)	53.20 ± 0.699	56.70 ± 1.21	59.43 ± 1.60^ab^	65.90 ± 2.05	Block 1
	GG(46)	54.83 ± 1.173	56.61 ± 1.22	59.53 ± 1.62^a^	65.80 ± 2.07	Block 1
g.6774 A > G	AA(34)	49.59 ± 1.437	55.69 ± 1.21^b^	58.28 ± 1.60^b^	64.79 ± 2.06^b^	Block 1
	AG(167)	53.18 ± 0.729	56.75 ± 1.18^a^	59.44 ± 1.58^a^	65.89 ± 2.04^a^	Block 1
	GG(265)	51.97 ± 0.628	56.53 ± 1.18^a^	59.23 ± 1.57^a^	65.64 ± 2.03^a^	Block 1
g.7088 G > A	AA(244)	52.64 ± 0.647	56.74 ± 1.24^a^	59.46 ± 1.73^a^	65.87 ± 2.11^a^	Block 1
	GA(165)	53.40 ± 0.716	56.87 ± 1.24^a^	59.60 ± 1.73a	66.05 ± 2.12^a^	Block 1
	GG(57)	48.63 ± 1.003	55.45 ± 1.25^b^	58.11 ± 1.74^b^	64.57 ± 2.13^b^	Block 1
g.7233 C > T	TT(424)	50.91 ± 1.150	56.65 ± 1.22	59.33 ± 1.62	65.79 ± 2.06	Block 1
	TC(42)	52.42 ± 0.570	56.50 ± 1.20	59.20 ± 1.60	65.63 ± 2.04	Block 1
g.7354 T > C	CC(267)	51.97 ± 0.628	56.52 ± 1.18^a^	59.22 ± 1.57^a^	65.62 ± 2.04^a^	Block 1
	CT(165)	53.18 ± 0.729	56.77 ± 1.18^a^	59.46 ± 1.58^a^	65.91 ± 2.04^a^	Block 1
	TT(34)	49.59 ± 1.437	55.69 ± 1.21^b^	58.28 ± 1.60^b^	64.79 ± 2.06^b^	Block 1
g.7437 T > G	GG(265)	51.97 ± 0.628	56.53 ± 1.18^a^	59.23 ± 1.57^a^	65.64 ± 2.03^a^	Block 1
	GT(167)	53.18 ± 0.729	56.75 ± 1.18^a^	59.44 ± 1.58^a^	65.89 ± 2.04^a^	Block 1
	TT(34)	49.59 ± 1.437	55.69 ± 1.21^b^	58.28 ± 1.60^b^	64.79 ± 2.06^b^	Block 1
g.7680 T > A	AA(287)	52.08 ± 0.627	56.65 ± 1.16^a^	59.32 ± 1.56^a^	65.78 ± 2.01^a^	Block 1
	TA(158)	52.74 ± 0.732	56.58 ± 1.16^a^	59.34 ± 1.56^a^	65.67 ± 2.02^a^	Block 1
	TT(23)	49.73 ± 1.858	55.44 ± 1.21^b^	57.84 ± 1.60^b^	64.68 ± 2.06^b^	Block 1
g.9312 T > A	AA(249)	52.67 ± 0.649	56.74 ± 1.23^a^	59.46 ± 1.72^a^	65.87 ± 2.11^a^	Block 1
	TA(160)	53.40 ± 0.731	56.85 ± 1.24^a^	59.58 ± 1.72^a^	66.05 ± 2.11^a^	Block 1
	TT(57)	48.91 ± 1.010	55.48 ± 1.24^b^	58.14 ± 1.73^b^	64.57 ± 2.12^b^	Block 1
g.11638 A > G	AA(33)	49.50 ± 1.460	55.90 ± 1.30^b^	58.52 ± 1.83^b^	64.80 ± 2.06^b^	Block 1
	AG(150)	53.86 ± 0.742	56.93 ± 1.28^a^	59.73 ± 1.82^a^	66.03 ± 2.05^a^	Block 1
	GG(246)	52.56 ± 0.639	56.81 ± 1.27^a^	59.54 ± 1.82^a^	65.72 ± 2.05^a^	Block 1
g.11846 A > G	AA(57)	48.63 ± 1.002	55.45 ± 1.25^b^	58.11 ± 1.74^b^	64.57 ± 2.13^b^	Block 1
	GA(159)	53.51 ± 0.726	56.87 ± 1.24^a^	59.60 ± 1.73^a^	66.05 ± 2.12^a^	Block 1
	GG(250)	52.64 ± 0.642	56.75 ± 1.24^a^	59.47 ± 1.73^a^	65.87 ± 2.12^a^	Block 1
g.12074 A > G	AA(34)	49.59 ± 1.435	55.71 ± 1.25^b^	58.27 ± 1.61^b^	64.78 ± 2.04^b^	Block 1
	AG(159)	53.40 ± 0.737	56.72 ± 1.22^a^	59.45 ± 1.59^a^	65.92 ± 2.01^a^	Block 1
	GG(272)	51.98 ± 0.624	56.47 ± 1.22^a^	59.21 ± 1.58^a^	65.63 ± 2.01^a^	Block1
g.12361 T > A	AA(247)	52.53 ± 0.644	56.73 ± 1.23^a^	59.44 ± 1.71^a^	65.84 ± 2.11^a^	–
	AT(163)	53.40 ± 0.724	56.83 ± 1.23^a^	59.56 ± 1.71^a^	66.06 ± 2.11^a^	–
	TT(59)	48.98 ± 0.989	55.54 ± 1.24^b^	58.20 ± 1.72^b^	64.65 ± 2.12^b^	–
g.13815 G > A	AA(266)	51.97 ± 0.628	56.53 ± 1.18^a^	59.23 ± 1.57^a^	65.64 ± 2.03^a^	Block 2
	GA(166)	53.18 ± 0.729	56.75 ± 1.18^a^	59.44 ± 1.58^a^	65.89 ± 2.04^a^	Block 2
	GG(34)	49.59 ± 1.437	55.69 ± 1.21^b^	58.28 ± 1.60^b^	64.79 ± 2.06^b^	Block 2
g.13963 A > T	AA(35)	49.81 ± 1.408	55.74 ± 1.21^b^	58.34 ± 1.60^b^	64.85 ± 2.06^b^	Block 2
	AT(168)	53.18 ± 0.729	56.74 ± 1.18^a^	59.43 ± 1.57^a^	65.88 ± 2.03^a^	Block 2
	TT(264)	51.97 ± 0.629	56.53 ± 1.18^a^	59.23 ± 1.57^a^	65.64 ± 2.03^a^	Block 2
g.21814 A > C	AA(22)	49.66 ± 1.928	55.64 ± 1.22^b^	58.03 ± 1.59^b^	64.83 ± 2.07	Block 2
	CA(151)	53.08 ± 0.754	56.60 ± 1.17^a^	59.33 ± 1.55^a^	65.71 ± 2.02	Block 2
	CC(296)	51.97 ± 0.613	56.58 ± 1.17^a^	59.29 ± 1.55^a^	65.71 ± 2.02	Block 2
g.26816 A > G	AA(306)	51.86 ± 0.609	56.58 ± 1.18^a^	59.26 ± 1.58^a^	65.66 ± 2.04	Block 2
	AG(140)	53.29 ± 0.774	56.56 ± 1.19^a^	59.32 ± 1.59^a^	65.75 ± 2.05	Block 2
	GG(20)	50.52 ± 2.018	55.63 ± 1.24^b^	58.01 ± 1.64^b^	64.77 ± 2.10	Block 2
g.26893 T > C	CC(401)	52.88 ± 0.576	56.74 ± 1.28	59.51 ± 1.83	65.73 ± 2.06	–
	CT(43)	51.12 ± 1.118	56.61 ± 1.30	59.34 ± 1.84	65.91 ± 2.07	–
g.27192 A > G	GG(452)	52.30 ± 0.556	56.50 ± 1.20	59.22 ± 1.59	65.68 ± 2.03	–
	GA(17)	49.39 ± 1.935	56.65 ± 1.26	58.99 ± 1.65	65.25 ± 2.10	–
g.29006 G > C	CC(120)	52.18 ± 0.796	56.18 ± 1.24^b^	59.08 ± 1.61^ab^	65.53 ± 2.05	Block 3
	CG(225)	52.29 ± 0.681	56.73 ± 1.23^a^	59.45 ± 1.62^a^	65.88 ± 2.05	Block 3
	GG(121)	52.12 ± 0.795	56.46 ± 1.24^ab^	59.02 ± 1.61^b^	65.44 ± 2.05	Block 3
g.35483 T > C	CC(63)	52.22 ± 1.010	56.40 ± 1.25^ab^	58.92 ± 1.63	65.34 ± 2.06	Block 3
	TC(206)	51.73 ± 0.695	56.73 ± 1.24^a^	59.39 ± 1.62	65.72 ± 2.05	Block 3
	TT(197)	52.63 ± 0.677	56.27 ± 1.24^b^	59.12 ± 1.62	65.66 ± 2.05	Block 3
g.38004 A > C	AA(4)	51.34 ± 4.474	56.07 ± 1.51	58.80 ± 1.87	63.65 ± 2.33	Block 3
	CA(75)	51.93 ± 0.995	56.48 ± 1.24	59.14 ± 1.61	65.68 ± 2.05	Block 3
	CC(387)	52.31 ± 0.577	56.48 ± 1.23	59.23 ± 1.60	65.65 ± 2.04	Block 3
g.40816 C > T	CC(199)	51.19 ± 0.657	56.32 ± 1.23^b^	58.99 ± 1.62^b^	65.37 ± 2.06^b^	Block 3
	TC(201)	53.09 ± 0.692	56.70 ± 1.23^a^	59.48 ± 1.62^a^	65.97 ± 2.06^a^	Block 3
	TT(66)	53.76 ± 1.011	56.59 ± 1.24^ab^	59.43 ± 1.63^ab^	65.94 ± 2.07^ab^	Block 3

Note: At the same loci, different superscript lowercase letters represent P < 0.05, and the same letter or no letters represent P > 0.05.

Among these body size-related SNPs, 15 were distributed across three linkage blocks. Intriguingly, a total of 10 SNPs, including g.6774 A > G, g.7088 G > A, g.7354 T > C, g.7437 T > G, g.7680 T > A, g.11638 A > G, g.12074 A > G in block 1, g.13815 G > A, g.13963 A > T in block 2, and g.40816 C > T in block 3 were significantly associated with BH, BL, and CC simultaneously. The g.21814 A > C, g.26816 A > G, and g.29006 G > C were significantly correlated with BH and BL (p < 0.05) (Table 3).

**Table 3.** Correlation between SNP loci in linked regions and growth traits in Nanjiang Yellow goat.

Block	Locus	Body weight(kg)	Body height(cm)	Body length(cm)	Chest circumference(cm)
Block1	g.5705 A > G	–	–	*	–
Block1	g.6774 A > G	–	*	*	*
Block1	g.7088 G > A	–	*	*	*
Block1	g.7354 T > C	–	*	*	*
Block1	g.7437 T > G	–	*	*	*
Block1	g.7680 T > A	–	*	*	*
Block1	g.11638 A > G	–	*	*	*
Block1	g.12074 A > G	–	*	*	*
Block2	g.13815 G > A	–	*	*	*
Block2	g.13963 A > T	–	*	*	*
Block2	g.21814 A > C	–	*	*	–
Block2	g.26816 A > G	–	*	*	–
Block3	g.29006 G > C	–	*	*	–
Block3	g.35483 T > C	–	*	–	–
Block3	g.38004 A > C	–	–	–	–
Block3	g.40816 C > T	–	*	–	*

Note: “+” Significantly related, “-” Indicates insignificant correlation.

The analysis of combined genotypes based on linked SNPs revealed that 29 genotype combinations showed no significant association with BW (Table 4). However, goats carrying specific genotypes combinations in Block 2 exhibited superior body size traits. For instance, individuals with the GGAAAAGG genotype displayed the largest BH (59.62 ± 0.67 cm) and BL (63.17 ± 0.81 cm), with a CC of 72.75 ± 1.16 cm, whereas those with the GGAACAAG genotype had the greatest CC (72.86 ± 1.26 cm) alongside relatively high BH (59.55 ± 0.73 cm) and BL (62.40 ± 0.87 cm). In contrast, goats with the CCTTCCCC genotype in Block 3 had the heaviest BW (21.92 ± 0.18 kg) but the smallest body dimensions, including BH (54.81 ± 1.31 cm), BL (57.16 ± 1.84 cm), and CC (63.60 ± 2.23 cm), compared with other Block 2 and 3 genotypes (Table 4). Collectively, these findings suggest that *NCAPG* polymorphisms influence body weight and size in a genotype- and distribution-dependent manner.

**Table 4.** Association analysis of combined genotype in linked regions with growth traits in Nanjiang Yellow goat.

Block	Combined genotype	Proportion/%	Body weight/kg	Body height/cm	Body length/cm	Chest circumference/cm
Block1	GAGGAACCGGAAGGGG(116)	27.42	22.02 ± 0.18	56.96 ± 1.26^abc^	59.71 ± 1.80^ab^	65.71 ± 2.05^bcd^
Block1	AAGGAACCGGAAGGGG(77)	18.2	22.01 ± 0.18	56.77 ± 1.26^bcd^	59.41 ± 1.80^bc^	65.82 ± 2.05^abc^
Block1	AAAGGACTGTTAAGAG(63)	14.89	22.04 ± 0.18	56.91 ± 1.27^abc^	59.81 ± 1.80^ab^	65.90 ± 2.05^abc^
Block1	GAAGGACTGTTAAGAG(57)	13.48	22.05 ± 0.18	57.14 ± 1.27^ab^	59.98 ± 1.81^ab^	66.31 ± 2.06^a^
Block1	GGGGAACCGGAAGGGG(43)	10.17	22.06 ± 0.18	56.89 ± 1.27^abc^	59.92 ± 1.81^ab^	65.84 ± 2.06^abc^
Block1	AAAAGGTTTTTTAAAA(22)	5.2	21.97 ± 0.19	55.87 ± 1.30^d^	58.38 ± 1.83^c^	64.63 ± 2.08^d^
Block1	AAAGGACTGTAAAGAG(17)	4.02	22.05 ± 0.19	57.52 ± 1.30^a^	60.19 ± 1.83^a^	66.30 ± 2.08^ab^
Block1	AAAAGGTTTTTAAAAA(11)	2.6	22.08 ± 0.19	56.12 ± 1.30 ^cd^	59.00 ± 1.85^bc^	65.18 ± 2.10 ^cd^
Block1	GAAGGACTGTAAAGAG(9)	2.13	22.04 ± 0.20	56.30 ± 1.33^bcd^	59.02 ± 1.86^bc^	65.75 ± 2.10^abcd^
Block1	GAAGGACTGTTAGGGG(4)	0.94	21.90 ± 0.22	56.83 ± 1.57^abcd^	59.57 ± 2.07^abc^	67.78 ± 2.32^a^
Block1	AAAGGACTGTTAGGGG(4)	0.94	22.04 ± 0.22	56.62 ± 1.40^bcd^	59.05 ± 1.92^bc^	64.80 ± 2.16 ^cd^
Block2	AATTCCAA(263)	56.8	21.82 ± 0.18	55.96 ± 0.55^c^	58.89 ± 0.68^e^	64.61 ± 1.00^d^
Block2	GAATCAAG(125)	27	21.86 ± 0.18	56.98 ± 0.58^bc^	59.71 ± 0.71^de^	67.83 ± 1.03^c^
Block2	GAATCCAA(28)	6.05	21.83 ± 0.18	58.24 ± 0.64^ab^	61.31 ± 0.77^c^	69.81 ± 1.12^b^
Block2	GGAAAAGG(20)	4.32	21.80 ± 0.19	59.62 ± 0.67^a^	63.17 ± 0.81^a^	72.75 ± 1.16^a^
Block2	GAATCAAA(13)	2.8	21.83 ± 0.19	57.12 ± 0.72^bc^	59.86 ± 0.86^d^	66.34 ± 1.25^c^
Block2	GGAACAAG(12)	2.6	21.88 ± 0.19	59.55 ± 0.73^a^	62.40 ± 0.87^b^	72.86 ± 1.26^a^
Block2	GGAAAAAG(2)	0.43	21.51 ± 0.25	58.08 ± 1.30^abc^	60.63 ± 1.50 ^cd^	70.34 ± 2.15^ab^
Block3	CGTCCCTC(125)	26.5	21.78 ± 0.17	56.93 ± 1.27^ab^	59.69 ± 1.81^ab^	66.00 ± 2.19^ab^
Block3	CCTTCCTT(66)	13.3	21.75 ± 0.17	56.80 ± 1.28^abc^	59.69 ± 1.82^ab^	66.13 ± 2.20^ab^
Block3	GGCCCCCC(63)	14	21.73 ± 0.17	56.45 ± 1.30^abc^	59.23 ± 1.81^bc^	65.61 ± 2.20^bc^
Block3	CGTTCCTC(50)	10.6	21.77 ± 0.18	57.08 ± 1.28^ab^	60.01 ± 1.82^a^	66.65 ± 2.20^a^
Block3	GGTCCCCC(48)	10.1	21.77 ± 0.17	56.84 ± 1.28^abc^	59.67 ± 1.82^ab^	65.97 ± 2.20^abc^
Block3	CGTCCACC(32)	6.7	21.80 ± 0.17	57.26 ± 1.29^a^	59.81 ± 1.83^ab^	66.00 ± 2.21^abc^
Block3	CCTTCATC(25)	5.3	21.75 ± 0.18	56.60 ± 1.28^bc^	59.12 ± 1.84^bc^	65.90 ± 2.22^abc^
Block3	CCTTCCCC(23)	4.8	21.92 ± 0.18	54.81 ± 1.31^d^	57.16 ± 1.84^d^	63.60 ± 2.23^d^
Block3	CGTTCACC(17)	3.6	21.81 ± 0.18	55.98 ± 1.32^c^	58.89 ± 1.85^bc^	65.70 ± 2.24^abc^
Block3	GGTTCCCC(10)	2.1	21.69 ± 0.19	55.89 ± 1.37 ^cd^	57.99 ± 1.90 ^cd^	64.54 ± 2.29 ^cd^
Block3	CCTTAACC(4)	0.8	21.83 ± 0.21	56.33 ± 1.52^bc^	59.16 ± 2.04^bc^	64.01 ± 2.45 ^cd^

Note: At the same block, different superscript lowercase letters represent P<0.05, and the same letter or no letters represent P>0.05.

## 4. Discussion

Growth traits are regarded as a highly economic feature in meat goat production and greatly interest researchers and farmers. It has been demonstrated that *NCAPG* controls muscle generation in sheep, which influences growth traits.^32^ This study investigated the genetic polymorphisms of the *NCAPG* gene and their potential associations with growth-related traits in Nanjiang Yellow goats in order to look into how these polymorphisms affect goat. The correlation research shows that the chest circumference of the Nanjiang Yellow goats, which might be a useful indicator for body weight estimation, has the strongest relationship with their body weight. This aligns with the findings of earlier research.^33^

The *NCAPG* gene of the Nanjiang Yellow goat population had 24 SNPs that were confirmed in this investigation. Among these, 19 SNPs agreed with Hardy–Weinberg equilibrium (P > 0.05). The discovery that 4 SNPs were found to be under selection pressure (P < 0.05), which is noteworthy since it suggests that the Nanjiang Yellow goat has experienced some artificial selection. Moreover, 20 SNPs exhibited moderate polymorphism, whereas 3 had low polymorphism indicating an intermediate genetic diversity for the *NCAPG* gene in the Nanjiang Yellow goat.

In the Nanjiang Yellow goat, this study discovered a g.5705 A > G mutation in exon 6 of the *NCAPG* gene, which causes an amino acid shift from Ile to Met. This mutation occurs in both YCG1 and PDS5 interaction domains, which are critical for condensin complex assembly. The substitution of Ile with Met results in the loss of hydrophobic interactions within the protein, leading to alterations in secondary structure and modifications in tertiary structure, thereby reducing protein stability. This structural alteration may reduce condensin activity during mitosis, delay chromosome segregation, and enhance proliferative signaling pathways, thereby prolonging the metaphase of growth plate stem cells. Ultimately, these changes contribute to variations in body size in goats.^24^^,^^25^^,^^34^^,^^35^ According to the protein prediction results, g.5705 A > G modifies local microstructure and domains of the protein but has little impact on its physico-chemical characteristics.

In addition, the 24 SNPs identified in Nanjiang Yellow goats were found to be either wild-type or mutant alleles in closely related species, indicating that these SNPs exhibit a relatively conserved pattern across related species. Interestingly, cattle also have this same amino acid change from Ile to Met, albeit it results from a c.1326 T > G mutation. The sheep also exhibit the same mutation at the g.7680 T > A locus. These findings reveal an intriguing evolutionary pattern of convergent amino acid substitution in NCAPG across ruminant species. While Nanjiang Yellow goats and cattle both exhibit an isoleucine-to-methionine change in the NCAPG protein, this results from distinct genomic mutations (g.5705 A > G in goats vs. c.1326 T > G in cattle), suggesting independent selection for similar molecular functions. Among these 24 SNP loci, 22 SNPs were located in intronic regions that inherently lack coding capacity but may regulate multiple traits through specific mechanisms. For instance, some non-coding SNPs could influence gene expression by altering transcription factors and chromatin structure.^36^^,^^37^

Nowadays, most research on association between *NCAPG* and growth traits has been done in humans, cattle, horses, poultry, and sheep.^38–41^ In this study, 24 SNPs within the *NCAPG* gene were noticed in Nanjiang Yellow goats, 19 of them were shown to have significant correlations with growth traits. Notably, a total of 10 SNPs were associated with BH, BL, and CC at the same time. It is reported that polymorphisms in the leptin (*LEP*), β-1,4-N-acetylgalactosamine transferase 2 (*B4GALNT2*), insulin-like growth factor I (*IGF-I*), and myocyte enhancer factor 2 (*MEF2*) genes were all intricately linked to the growth performance of Nanjiang Yellow goats.^42–45^ These studies suggest that the growth traits in Nanjiang Yellow goat are complicated attributes linked to multiple genes, and more research is needed to determine how *NCAPG* affects growth traits.

Multiple livestock species have provided ample evidence for the critical role of the *NCAPG* gene in controlling growth-related parameters. The *NCAPG* gene significantly influences body weight, longissimus muscle development, carcass traits, and body frame size in cattle.^11^^,^^24^^,^^25^^,^^35^ The c.1326 T > G of *NCAPG* is the identified QTL potentially affecting divergent bovine fetal growth.^35^ Similarly, a correlation between *NCAPG* and rapid growth as well as increased body size has been observed in donkeys through genome-wide selective scanning.^21^^,^^46^ In sheep, a genome-wide scan has also identified an association between the *NCAPG* gene and traits related to body size, growth, and feed intake.^47^^,^^48^ Furthermore, the *LCORL/NCAPG* gene exhibits a significant correlation with body height in horses,^22^^,^^49^ while the NCAPG-LCORL locus represents a primary overlap zone for QTL associated with body length in pigs^50^. These findings strongly support the notion that the *NCAPG* gene is closely linked to body size traits in livestock and poultry. Future studies on regulatory mechanisms of function verification of *NCAPG*, such as g.5705 A > G (NCAPG-281 I > M) in exon 6 and g.7088 G > A (in the selected state and distributed in block 1), are therefore strongly recommended.

## Conclusions

In summary, this study identified 24 SNPs within the *NCAPG* gene in Nanjiang Yellow goats, among which several were significantly associated with growth traits such as body height, body length, and chest circumference. Notably, the g.5705 A > G mutation in exon 6, predicted to reduce protein stability, represents a promising candidate variant potentially underlying body size variation. The observed conservation of these polymorphisms across ruminant species further underscores the evolutionary importance of *NCAPG* in growth regulation. While these findings provide valuable genetic markers that could support marker-assisted selection, it is essential to acknowledge that functional validation—particularly of mutations like g.5705 A > G—is required before their incorporation into breeding strategies. Future work integrating gene expression assays, protein functional analyses, and large-scale association studies will be crucial to establish causality and to fully harness the potential of *NCAPG* polymorphisms in goat genetic improvement programs.

## Supplementary Material

Table S1.xls

Figure S3. The hydrophobicity of NCAPG-WT and NCAPG-I281M_01.jpg

Table S6.xls

Figure S4. Effect of the NCAPG SNP mutations on protein phosphorylation sites_01.jpg

Figure S2. The linkage disequilibrium map of NCAPG SNPs in Nanjiang Yellow goat_01.jpg

Table S5.xls

Figure S1. Conserved domains on NCAPG_01.jpg

Table S3.xls

Table S2.xls

Table S4.xls

## Data Availability

The *NCAPG* gene reference sequence is downloaded from the NCBI (https://www.ncbi.nlm.nih.gov/) with sequence number: NC_030813.1.
