# Supplementary figures and images for: Polymorphism of NCAPG gene and its association with growth traits in Nanjiang Yellow goat
Source: Anim Biotechnol. 2025 Oct 7;36(1):2565161. doi: 10.1080/10495398.2025.2565161 (PMC12674292; doi:10.1080/10495398.2025.2565161)

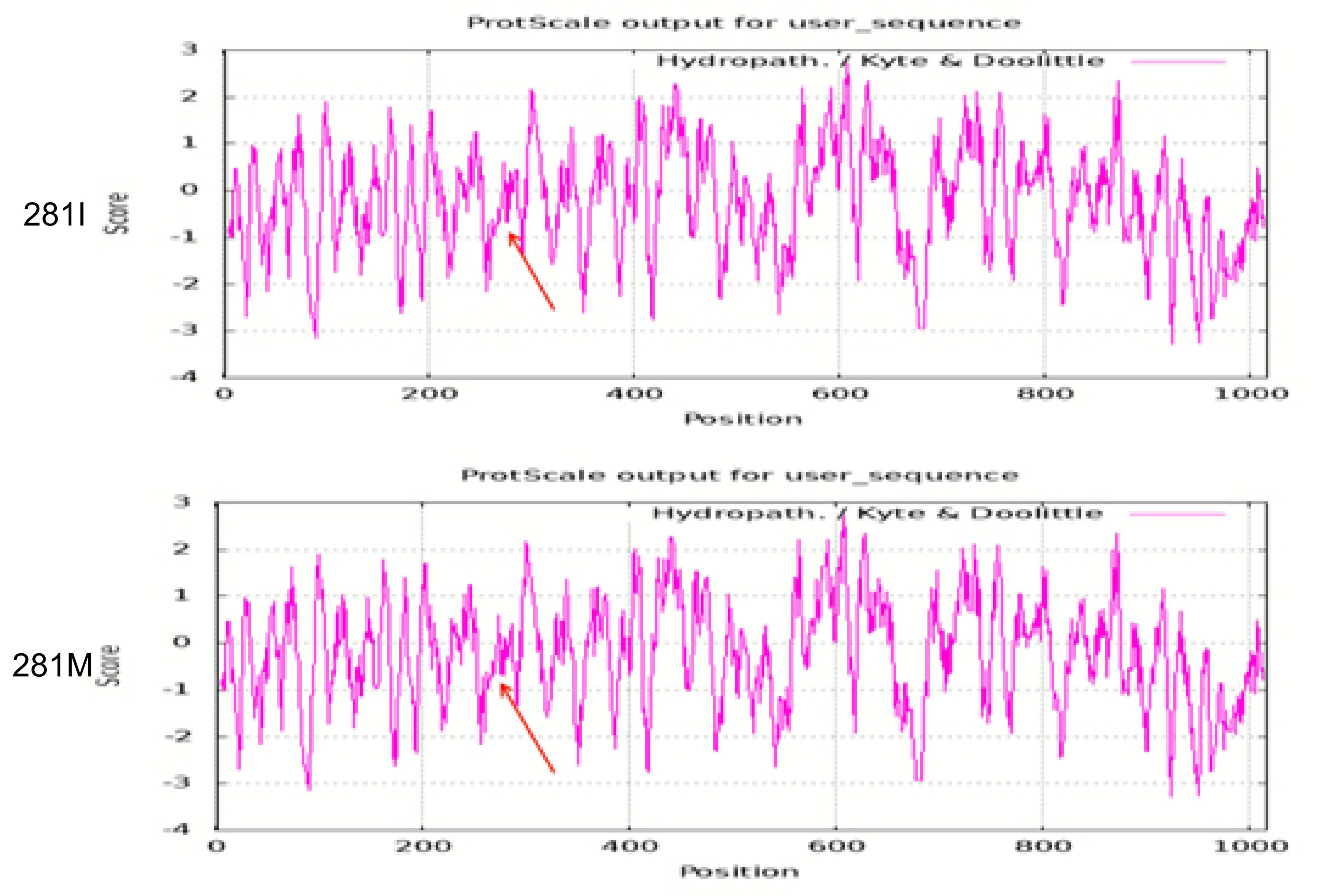

Supplement: Figure S3. The hydrophobicity of NCAPG-WT and NCAPG-I281M_01.jpg [file LABT_A_2565161_SM1331.jpg]

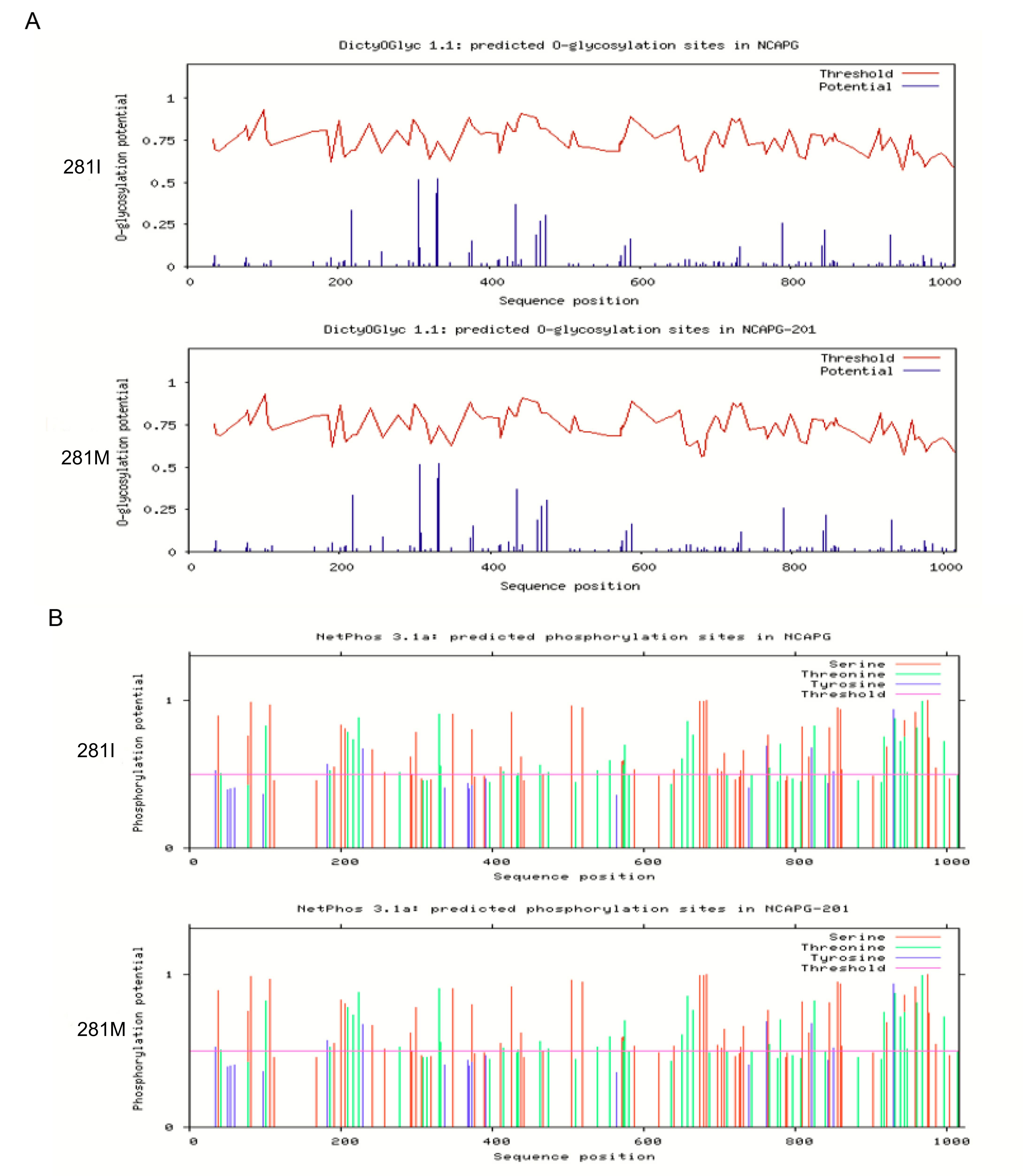

Supplement: Figure S4. Effect of the NCAPG SNP mutations on protein phosphorylation sites_01.jpg [file LABT_A_2565161_SM1329.jpg]

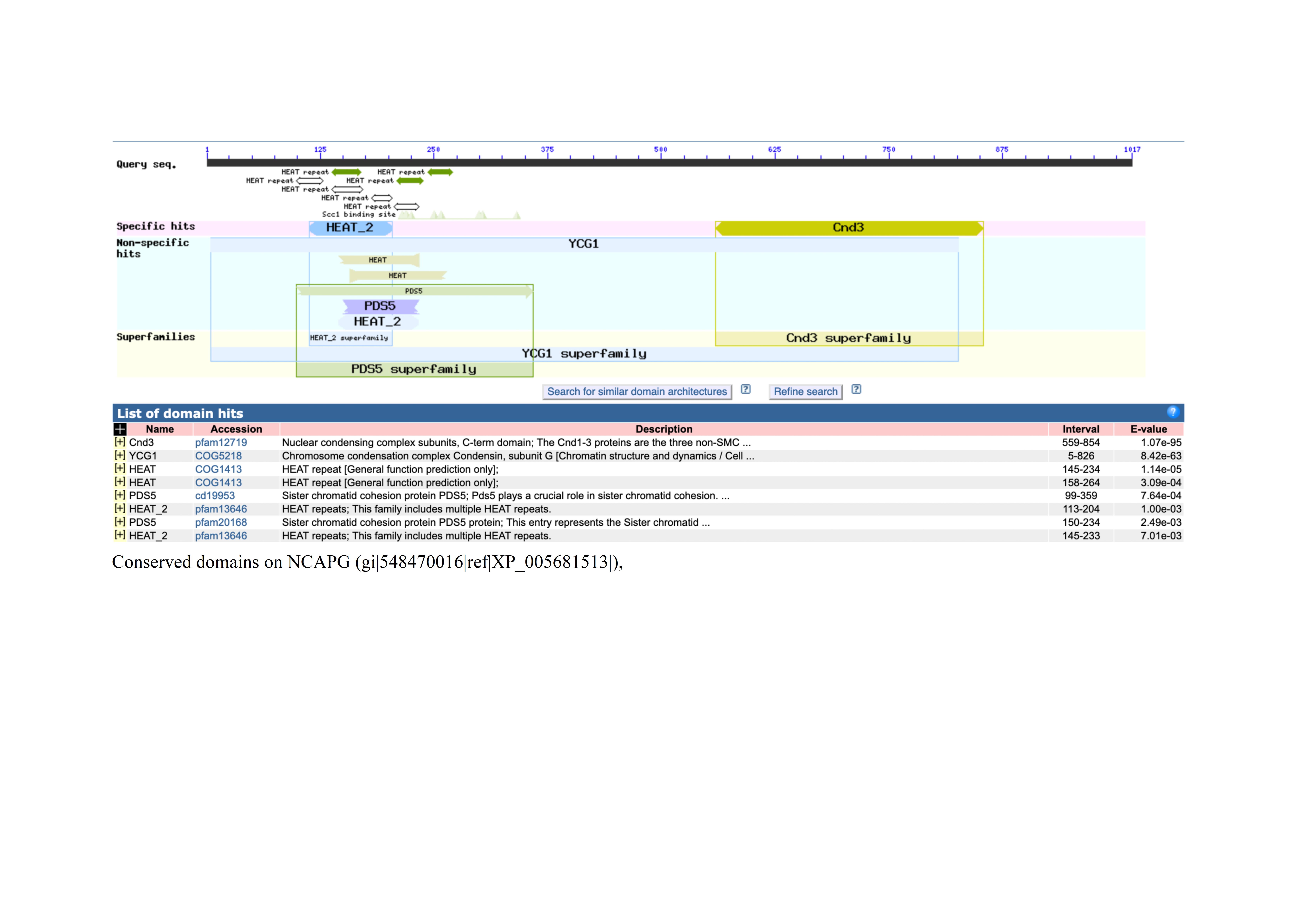

Supplement: Figure S1. Conserved domains on NCAPG_01.jpg [file LABT_A_2565161_SM1326.jpg]
